# Supplementary material for: Developmental Transformation and Reduction of Connective Cavities within the Subchondral Bone
Source: Int J Mol Sci. 2019 Feb 12;20(3):770. doi: 10.3390/ijms20030770 (PMC6387253; doi:10.3390/ijms20030770)
Supplement: Supplementary file 1 [file ijms-20-00770-s001.pdf]

Experimental method: Bone mineralization measurement

The region of interest for mineralization measurements was a grid with 10 x 8 individual rectangles. Each row corresponded to a certain depth separated by 100 μm intervals. In each of the rectangles, a measuring field of 6 × 6 pixels was manually positioned on the bone structure. Care was taken so that the position of the measuring field would not be at the edge of the bony structure to avoid partial volume effects. In the marginal areas, a bone, as well as a hollow area can be represented in one pixel simultaneously. The evaluation of such areas can lead to incorrectly low mineralization measurement values. This phenomenon is called partial volume effect; furthermore, very small bone cavities, under the resolution limit, can strongly alter the distribution of mineralization. The final mineralization value attributed to each depth is the mean ± SD of the 10 rectangles for 3 samples/group. In order to limit measurement errors based on the partial volume effect, only measurements with a standard deviation of less than 150 mg HA/cm3 were used.

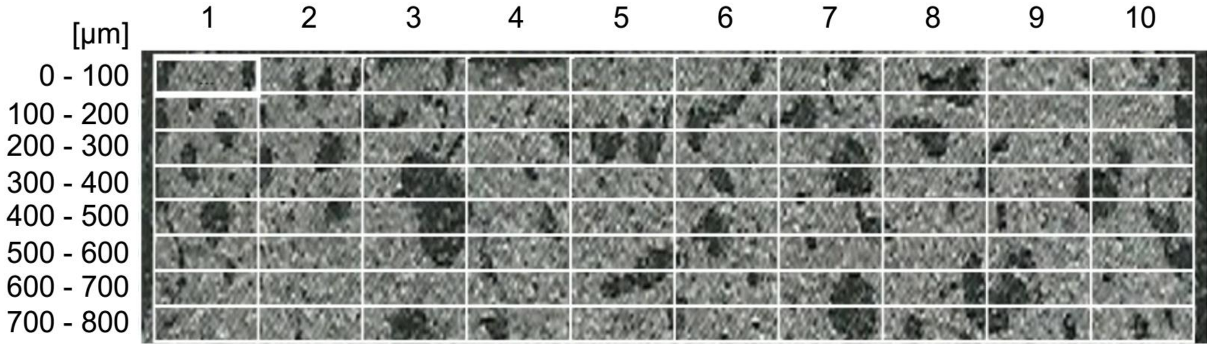

Supplementary Figure S1. The region of interest for mineralization measurements.

Statistics: The correlation between chemical composition and distance from the tidemark

A correlation study was performed to assess whether an increase or decrease in bone's chemical contents/ratios correspond to an increase or decrease in the distance from the tidemark. When the statistical analysis was conducted for the whole subchondral zone (700 μm) no significant correlation was observed. Upon further observations, however, we detected two regions that seemed to exhibit different behaviors: 1) 0-300 μm containing five EDX measurement areas (0-50, 50-100, 100-150, 150-200, and 200-300 μm), and 2) 300-700 μm, containing four measurement areas (300-400, 400-500, 500-600, 600-700 μm). Thus, a linear regression model for individual chemical compositions/ratios was fitted into each of these two areas. The significance is measured based on the level that the slope is different from zero. Results of this analysis revealed significant positive correlations ($0.88 < r < 0.98$) for Ca and P contents in the 0-300 μm region. The pattern was almost identical for both calves and cattle, with insignificant changes after the 300 μm distance from the tidemark. In cattle, the C/P ratio was positively correlated with depth in the 300-700 μm region. We also found that the Ca/P ratio did not significantly vary based on the depth from the tidemark despite the fact that both Ca and P showed depth-dependent behaviors.

Supplementary Table S1. Statistical correlation between chemical composition and distance from the tidemark in bovine bone.

Chemical Content/Ratio	Sample Type	Depth	Intercept	Pearson's r	*p*
C [%]	Calves	0-300 [µm]	26.39	-0.18	0.77
		300-700 [µm]	26.67472	-0.32374	0.67626
	Cattle	0-300 [µm]	24.19875	0.71078	0.17839
		300-700 [µm]	24.79778	0.80013	0.19987
Ca [%]	Calves	0-300 [µm]	23.67	0.88	*** 0.045**
		300-700 [µm]	24.29979	-0.62918	0.37082
	Cattle	0-300 [µm]	24.48015	0.93823	*** 0.01826**
		300-700 [µm]	24.78601	0.89774	0.10226
P [%]	Calves	0-300 [µm]	14.75	0.98	**** 0.003**
		300-700 [µm]	15.08543	0.03824	0.96176
	Cattle	0-300 [µm]	14.93755	0.97117	**** 0.00585**
		300-700 [µm]	15.21708	-0.30443	0.69557
C/Ca	Calves	0-300 [µm]	1.11	-0.41	0.49
		300-700 [µm]	1.09782	-0.17946	0.82054
	Cattle	0-300 [µm]	0.98827	0.46032	0.43532
		300-700 [µm]	1.00048	0.11789	0.88211
C/P	Calves	0-300 [µm]	1.78	-0.47	0.43
		300-700 [µm]	1.76825	-0.32823	0.67177
	Cattle	0-300 [µm]	1.61991	0.41784	0.48391
		300-700 [µm]	1.62959	0.98665	*** 0.01335**
Ca/P	Calves	0-300 [µm]	1.6	-0.06	0.92
		300-700 [µm]	1.61082	-0.62717	0.37283
	Cattle	0-300 [µm]	1.63911	-0.10656	0.86458
		300-700 [µm]	1.62882	0.81477	0.18523
